# Positive toxicology and reactive serology in tissue donors: a retrospective study over a 3-year period

**DOI:** 10.1007/s10561-020-09827-2

**Published:** 2020-04-03

**Authors:** Ellen Heck, Kristel Gruslin, Valerie Corder, W. Matthew Petroll, Jill Urban

**Affiliations:** 1 University of Texas Southwestern Medical Center, Dallas, TX USA; 2 Institute of Forensic Sciences Dallas County, Dallas, USA

**Keywords:** Positive toxicology, Reactive serology, Potential tissue donors, Correlation of data

## Abstract

Assessment of donor suitability and criteria development for tissue donation evaluation which appropriately addresses the risk factors for disease transmission, especially high risk for Hepatitis B or C, HIV or other transmissible diseases as defined by the Food and Drug Administration, FDA, is a continuing concern for tissue banks. The relationship of drug use, especially IV drugs, has been determined to be associated with an increased possibility of reactive serology (Centers for Disease Control and Prevention (USCDC) in Division of Viral Hepatitis, National Center for HIV/AIDS, Viral Hepatitis, STD, and TB Prevention. Hepatitis C questions and answers for health professionals. https://www.cdc.gov/hepatitis/hcv/hcvfaq.htm; Centers for Disease Control and Prevention (USCDC) in infectious diseases, opioids and injection drug use, 2018. https://www.cdc.gov/pwid/opioid-use.html; HIH National Institute on Drug Abuse in Health Consequences of Drug Misuse, 2017. https://www.drugabuse.gov/related-topics/health-consequences-drug-misuse). Therefore, prior drug use determined by medical social history screening frequently results in deferral of a potential donor even when the route of drug administration has not been determined to be intravenous. Because of the association of drug use in numerous cases, which come under Medical Examiner jurisdiction, a possible rule out of a number of otherwise suitable medical examiner cases could occur. This retrospective review of medical examiner cases, tissue bank referrals and tissue donors in a 3-year period examines the relationship, if any, between reactive serology and positive toxicology results. These results would appear to indicate assessment of donor medical social history screening is effective in reducing recovery of high-risk donors.

## Introduction

A retrospective examination of records of potential tissue referrals and recovered donors for a 3-year period, 2015–2017, were examined for the use of drugs as determined by toxicology screening and the finding of reactive serologies in this large urban medical examiner tissue donor population. Since these donations had proceeded prior to any toxicology results or reactive serologies, the medical social history information for these donors failed to indicate a potential for high risk for communicable disease. There were 318 donors in this category of medical examiner potential donors out of 843 total donors during the time period.

## Methods

Donor medical social history was obtained utilizing the standardized form developed by the American Association of Tissue Banks, AATB, (American Association of Tissue Banks 2014) and conducted by trained screeners with medical backgrounds. Screening, testing and medical records were further examined by medically trained individuals and all reviews of relevant documents completed before any tissue could be moved from quarantine for potential transplantation. When serologic testing proved reactive in any of the required tests, the tissue was discarded. Routine serology testing utilizing FDA approved test kits was conducted for HIV 1/HCV/HBVNAT, HIV1/2 plus O antibody, HCV antibody, Hepatitis B antigen, Hepatitis Bc antibody, West Nile virus, Syphilis RPR and Hepatitis C antibody by an accredited reference laboratory. Center for Disease Control and Prevention (USCDC) Division of Viral Hepatitis, National Center for HIV/AIDS, Viral Hepatitis, STD and TB Prevention. Hepatitis C Questions and Answers for Health Professionals. https://www.cdc.gov/hepatitis/hcv/hcvfaq.htm. Toxicology was performed utilizing standard methodology of ELISA, liquid chromatology and high-resolution mass spectrometry (Smith et al. 2007) which detects the presence of a variety of drugs including opioids, cannabis, codeine and methamphetamine. Toxicology tests are designed to identify and quantitate a wide variety of substances and metabolites including abused drugs and clinical drugs. The general drug screen provides comprehensive screening of biological fluids for several hundred drugs and metabolites. Once identified, the presence of these drugs is confirmed and quantitated. An ELISA screen detects opiates, cocaine, cannabinoids, benzodiazepines, barbiturates, phencyclidine and amphetamines, which are then confirmed by liquid chromatography and mass spectrometry. An alkaline screen (gas chromatography/mass spectrometry) is also used to identify cocaine, amphetamine, some opiates, benzodiazepines, and phencyclidine. These drugs can then be quantified with gas chromatography/flame ionization detection. An acid/neutral drug screen by gas chromatography/mass spectroscopy detects muscle relaxants. Volatiles such as diflouroethane are detected by head gas chromatography. Synthetic cannabinoids and “designer drugs” (such as U–47700) are sent out to a reference laboratory (NMS laboratories) for identification. The toxicology testing laboratory is accredited by the American National Standards Institute.

A random 3-month review in each of the 3 years of donor referrals was also performed to evaluate non-suitability for donation based on only medical social risk assessment. The donor population is composed of 104 donors in year 2015 ranging in age from 3 months to 71 years. In 2016, 124 donors ages 2 years to 71 years and in 2017, 2 months to 69 years for the 90 donors. The composition of this distribution can be seen in figure one. The male to female distribution for the 3-year period was 211 male and 107 female.

## Results

**Fig. 1:**
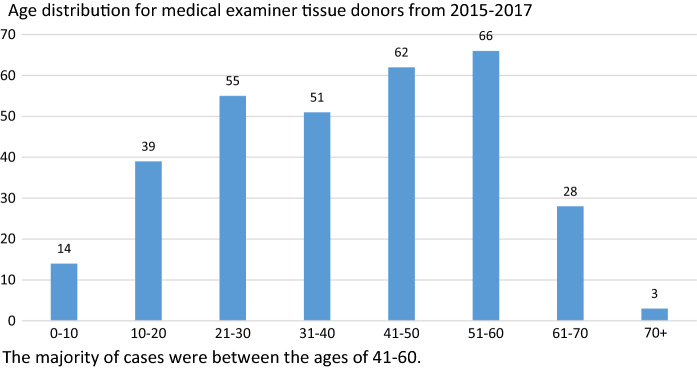
Age distribution for medical examiner tissue donors from 2015 to 2017. The majority of cases were between the ages of 41–60

Of the 104 cases reviewed in 2015, four were found to have one or more reactive serologies as seen in Table 1. Donor A was reactive for HBc, and HBV NAT. Donor B was reactive for HBc. Donor C was reactive for RPR (STD), and Donor D was reactive for HBC. Two of these serology reactive donors, A and B, had no toxicology findings. Toxicology, for the 104 cases, was positive in 49 individuals. In 2016, there were two reactive serologies and 61 positive toxicology screens in the 124 potential donors. For the 2016 two reactive serologies, Table 4, Donor E was reactive for HBVab, HBc Total and HCV NAT. Donor F was reactive for HBc only. Both donors had positive toxicology screens, Table 4. Five reactive serologies with 45 positive toxicology screens in 2017 were found for the 90 potential donors. Donor G was reactive for HBsAg, Donor H HCVAb, HBc Total; Donor I HBc Total; Donor J HCV Ab; and Donor K HBc Total. The list of toxicology findings can be seen in Table 2 and the drugs cannot differentiate between prescribed drugs and possible illicit drug administration. Of course, some of the drugs found on toxicology screening could be either prescribed for medical reasons or used for non-medical purposes, as may be suspected for heroin, cocaine and methamphetamine. The donor population was consistent with the overall age and gender of the non-medical examiner cases with 66.4% male to 33.6% female. Age distribution can be seen in Fig. 1 with the largest number between ages 41 and 60. It is important to note that during the same time periods, potential donors were consistently ruled out or deferred due to medical history screening prior to any tissue recovery. In a random sampling of data for 3 month periods in each of the 3 years, between approximately 12–30% of referrals were deferred for potential high risk by medical social history interview as seen in Table 3. The toxicology findings compared to the serology reactivity can be seen in Table 4. Four of the eleven serology reactive donors had no toxicology finding. One had only THC and alcohol. The alcohol alone is not generally considered to be an increased risk factor although liver damage may be a consideration (Seitz et al. 2018; Lucey et al. 2009). THC has no history of injection and might only be a consideration for other drug usage, although this has not been sufficiently documented (Secades-Villa et al. 2015). A comparison of each donor’s reactive serology with their identified toxicology panel can be seen in Table 4. Results for comparison of medical examiner data and total population serology and medical examiner serology and toxicology results were examined using Chi square analysis (see results Table 1).

**Table 1:** Summary of reactivity in medical examiner tissue donors from total tissue donors in a 3-year period

Year	Total tissue donors	Total tissue donor reactive serology	Medical examiner tissue donors	Medical examiner reactive serology	Positive medical examiner toxicology*
2015	298	12 (4%)	104	4 (3.8%)	49 (47%)
2016	299	11 (3.7%)	124	2 (1.6%)	61 (49.2%)
2017	246	12 (4.9%)	90	5 (5.5%)	45 (50%)
3 Year total	843	35	318	11	155

*Toxicology available for Medical Examiner donors only, not routinely performed by hospitals

Chi square = 166.695 with 1 degree of freedom (*P* ≤ 0.001) There is a significant difference in the percent of positive serology vs positive toxicology (i.e. higher percentage of donors have positive toxicology)

Chi square = 0.138 with 1 degree of freedom. (*P* = 0.711): No significant difference between serology results in “total donor” and Medical Examiner Donor samples

**Table 2:** Drugs found during toxicology testing for medical examiner tissue donors from 2015 to 2017

5F-AMB*	AKB-48-N*	Alprazolam	Amphetamine
Carisoprodol	Chlordiazepoxide	Clonazepam	Cocaine
Codeine	Cyclobenzaprine	Diazepam	Difluoroethane
Ethanol	Etizolam	Fentanyl	Heroin
Hydrocodone	Ketamine	Lorazepam	Meprobamate
Methadone	Methamphetamine	Methylphenidate	Morphine
Oxycodone	Phenobarbital	Temazepam	THC (tetrahydrocannabinol)
Tramadol	U-47700 (opioid analgesic)	Zolpidem	

*Indicates drug is a synthetic cannabinoid

**Table 3:** Random 3-month high risk medical-social history screening rule-outs of medical examiner tissue referrals and tissue donors

Time period	Total tissue referrals	Recovered tissue donors	High risk rule out (Med-Soc Hx)	Remaining referrals
April-June 2015	228	33 (14.5%)	27 (11.8%)	168
July-Sept 2016	229	28 (12.2%)	31 (13.5%)	170
Jan-March 2017	185	28 (15.1%)	27 (14.6%)	130

The 168 referrals in 2015, 170 in 2016 and 130 in 2017 did not become donors either because of lack of consent or first person authorization, medical-social history unrelated to high risk issues i.e.cancer, physical condition, inability to contact next of kin, and time of death to notification

**Table 4:** Serology and toxicology results for the 11 donors with reactive serology in medical examiner tissue donors from 2015-2017

Donor	Reactive serology results	Toxicology results
A	HBc total, HBV NAT	None
B	HBc total	None
C	RPR(STS)	THC
D	HBc total	Cocaine
E	HCV Ab, HBc total, HCV NAT	Amphetamine/methamphetamine
F	HBc total	Ethanol 0.02, THC
G	HBsAg	None
H	HCV Ab, HBc total	Tramadol, hydrocodone
I	HBc total	Chlordiazepoxide, diazepam
J	HCV Ab	Phenobarbital, morphine
K	HBc total	None

Four donors with reactive serology had negative toxicology findings. These donors were two in 2015 and two in 2017

## Discussion

The value of second hand medical social history screening is often debated as to its accuracy and value in the donation process (Solves et al. 2014; Fishman et al. 2012; Greenwald et al. 2012). This review would seem to indicate that very few high risk donors are recovered because of lack of risk identification during medical social history screening. Perhaps it is more likely that more than necessary are actually deferred out of caution for missing of risk. Though regrettable, if true, is nonetheless a foreseeable circumstance in a necessary effort to assure maximum safety for recipients. The percentage of reactive serology in this medical examiner population ranged between 2 to 5% while the deferral percentage ranged between 12 to 15%. In addition to the possible high risk deferrals, potential donors may not be realized due to a variety of factors which may include medical non high risk history i.e. cancer, age, time of the death notification, inability to contact next of kin or authorized historian, and physical condition. Fortunately, with donor registries in most states and increased education of the benefits of donation to the health care of others, tissue availability is no longer in short supply as it was in previous years (National Donate Life Month Registry Overview Report 2019). However, with increasing new and spreading viral diseases (Morens et al. 2013; Ebola virus disease-fact sheet October 18; Centers for Disease Control and Prevention (USCDC) 2018a, b), the appropriate review of criteria for donor risk screening must always be a paramount part of making transplantable tissues available and the balance between risk criteria assessed as conditions and practices change.

## References

[CR1] American Association of Tissue Banks (2014) Uniform donor risk assessment interview. https://www.aatb.org/standards/uniform-drai. Accessed 25 Aug 2014.

[CR2] Centers for Disease Control and Prevention (USCDC) (2018a) Infectious Diseases, Opioids and Injection Drug Use. https://www.cdc.gov/pwid/opioid-use.html. Accessed 19 July 2018

[CR3] Centers for Disease Control and Prevention (USCDC) (2018b) National Center for Emerging and Zoonotic Infectious Diseases, Division of Vector-Borne Diseases. December 10, 2018

[CR4] Centers for Disease Control and Prevention (USCDC) Division of Viral Hepatitis, National Center for HIV/AIDS, Viral Hepatitis, STD, and TB Prevention. Hepatitis C Questions and Answers for Health Professionals. https://www.cdc.gov/hepatitis/hcv/hcvfaq.htm. Accessed 18 Oct 2018

[CR5] Ebola Virus Disease-Fact Sheet. https://www.who.int/en/news-room/fact-sheets/detail/ebola-virus-disease. Accessed Oct 2008

[CR6] Fishman Jay A, Greenwald Melissa A, Grossi Paolo A (2012). Transmission of infection with human allografts: essential considerations in donor screening. Clin Infect Dis.

[CR7] Greenwald MA, Kuehnert MJ, Fishman JA (2012). Infectious disease transmission during organ and tissue transplantation. Emerg Infect Dis.

[CR8] HIH National Institute on Drug Abuse (2017) Health consequences of drug misuse. March 2017 https://www.drugabuse.gov/related-topics/health-consequences-drug-misuse

[CR9] Lucey MR, Mathurin P, Morgan TR (2009). Alcoholic hepatitis. N Engl J Med.

[CR10] Morens DM, Folkers G, Fauci AS (2013). The challenge of emerging and re-emerging infectious diseases. Nature.

[CR11] National donate life month registry overview report (2019). January 16 2019

[CR12] Secades-Villa R, Garcia-Rodríguez O, Jin CJ, Wang S, Blanco C (2015). Probability and predictors of the cannabis gateway effect: a national study. Int J Drug Policy.

[CR13] Seitz HK, Bataller R, Cortez-Pinto H (2018). Alcoholic liver disease. Nat Rev Dis Prim.

[CR14] Smith ML, Vorce SP, Holler JM (2007). Modern instrumental methods in forensic toxicology. J Anal Toxicol.

[CR15] Solves P, Mirabet V, Alvarez M (2014). Hepatitis B transmission by cell and tissue allografts: How safe is safe enough?. World J Gastroenterol.

